# Ultrasound-Guided Endometrial Polypectomy Performed in an Office Setting Without Hysteroscopy

**DOI:** 10.7759/cureus.107420

**Published:** 2026-04-20

**Authors:** Vijaya B Bayyarapu, Sirisha R Gundabattula, Pranuthi Rangaram, Sarah Ashrafi

**Affiliations:** 1 Department of Gynecology, Fernandez Hospital, Hyderabad, IND

**Keywords:** ambulatory gynecology, endometrial polyp, laparoscopic grasper, office gynecology, ultrasonography, uterine polypectomy

## Abstract

Endometrial polyps are localized overgrowths of the endometrium that protrude into the endometrial cavity. Symptomatic persistent endometrial polyps warrant removal. Hysteroscopic polypectomy is the gold standard for endometrial polyp removal, with office-based procedures gaining popularity in recent years. We report an alternative technique of endometrial polypectomy in the office under transabdominal ultrasound guidance using an atraumatic 5-mm laparoscopic grasper, which is particularly useful when a hysteroscope is unavailable. This procedure could be successfully completed in more than 95% of women within a median duration of 15 minutes, with a median pain score of 3 on a scale of 10. The advantages of a laparoscopic grasper over the oft-used ovum forceps or sponge-holding forceps are also highlighted. Ultrasound-guided outpatient polypectomy appears to be a safe, well-tolerated, and cost-effective alternative to hysteroscopy for endometrial polypectomy and warrants further research.

## Introduction

Endometrial polyps are focal proliferations that can occur anywhere in the uterine cavity. They are commonly encountered in gynecological practice and may be diagnosed in women of reproductive age as well as after menopause. Although the diagnosis may be incidental, its significance is in the setting of abnormal uterine bleeding, subfertility, and a potential risk of malignancy in certain clinical situations. Endometrial polyps are encountered more frequently after menopause, with an incidence being 12% in postmenopausal women vs. 6% in premenopausal women [1,2].

The majority of polyps detected on ultrasound are treated surgically, and the standard method for removing persistent or symptomatic endometrial polyps is hysteroscopy. It is the preferred approach, as blind curettage may miss small polyps. Facilitating complete resection minimizes recurrence. This procedure is almost always successful when performed under anesthesia; however, there may be risks such as uterine perforation and fluid overload [3]. According to the Global Community of Hysteroscopy Guidelines Committee [4], transvaginal ultrasound examination (with Doppler, 3D, and contrast instillation when necessary) is accurate for diagnosing endometrial polyps. Hysteroscopic polypectomy is safe with negligible risk of intrauterine adhesions and is recommended in symptomatic patients, postmenopausal women with incidentally diagnosed polyps larger than 2 cm, and in asymptomatic women with risk factors for endometrial cancer. Office hysteroscopy does not require admission, is more convenient, and is cost-effective than inpatient hysteroscopy.

At the study institute, hysteroscopic polypectomy under anesthesia is the standard treatment for endometrial polyps. Necessity is the mother of invention, and the restrictions imposed on elective surgeries during the COVID-19 pandemic in 2020 prompted the search for an alternative nonhysteroscopic office procedure. A literature search at the time revealed very few reports on ultrasound-guided polypectomies [5,6], and a novel technique for endometrial polyp retrieval was developed and fine-tuned.

## Technical report

**Figure 1:**
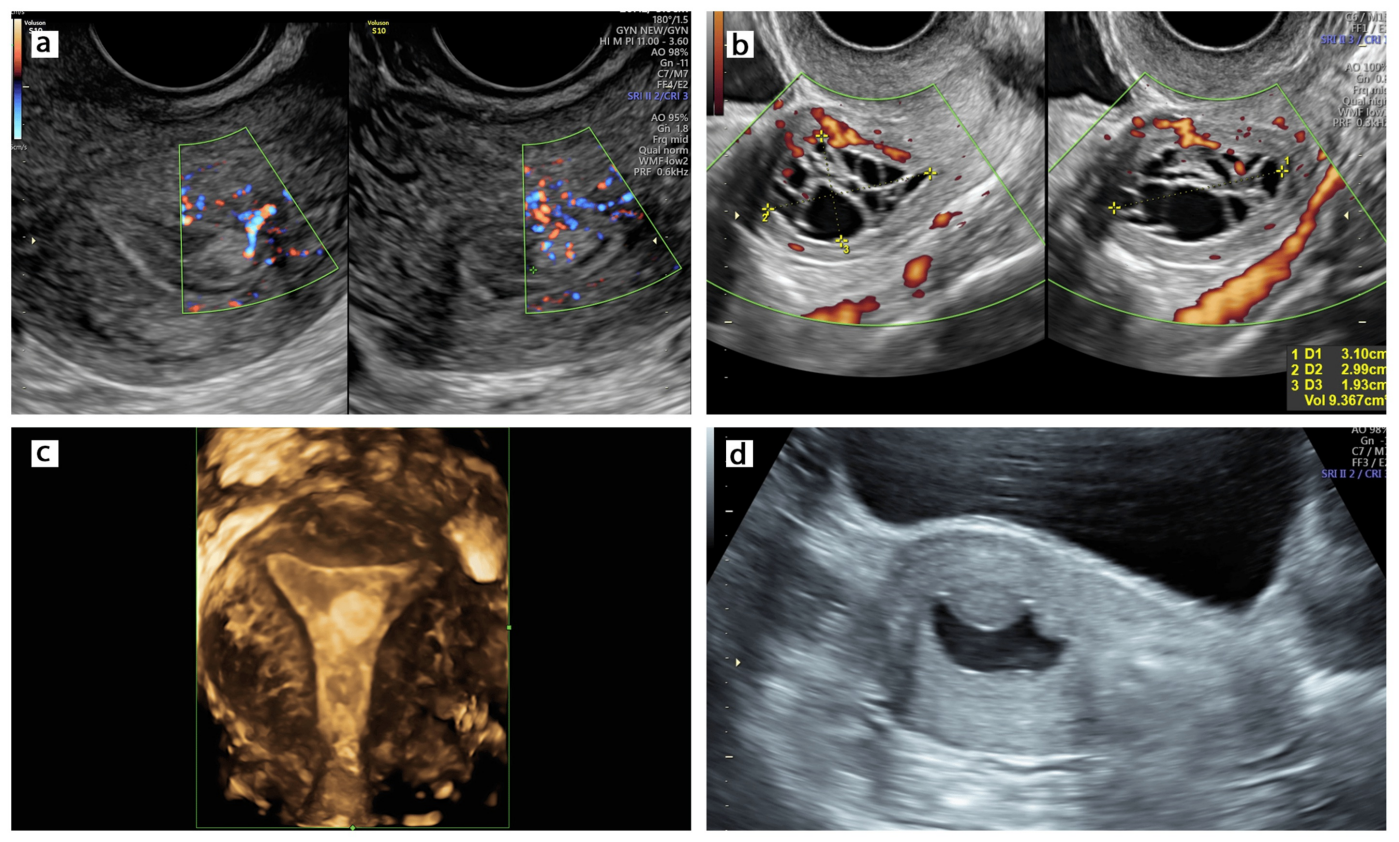
Ultrasound images of endometrial polyps. (a) Hyperechoic lesion with a feeder vessel on Doppler. (b) Polyp in a postmenopausal woman showing multiple cystic spaces. (c) 3D image of a polyp. (d) Polyp at saline sonography

Beginning in May 2020, this outpatient procedure was offered to women diagnosed with endometrial polyps using standard criteria [7] by gynecologists formally trained in pelvic ultrasound. They typically appear as hyperechoic lesions with a bright edge and a feeder vessel on Doppler examination (Figure 1). Only those women who did not consent to an office procedure were excluded. After obtaining informed consent, an analgesic combination of mefenamic acid 250 mg and dicyclomine 10 mg was given half an hour prior to the procedure. Women with a pinhole os were advised 200 mcg vaginal misoprostol an hour before polypectomy. A partly filled urinary bladder facilitated transabdominal ultrasound guidance. A Cusco speculum was used to visualize the uterine cervix, and the polyp was grasped, twisted, and retrieved using a 5-mm laparoscopic atraumatic grasper. This was repeated until the uterine cavity was confirmed to be empty (Video 1). The cervix was held with an Allis forceps for stabilization only if necessary. Gentle traction was applied on the posterior cervical lip if required to straighten the uterocervical axis in patients with retroverted uteri. Ultrasound assessment (with saline infusion when needed) was used to ascertain complete removal of the polyp. Failed entry into the uterine cavity or inability to retrieve the polyp in its entirety were documented.

**Video 1:** Ultrasound-guided endometrial polypectomy

The procedure was attempted in 99 women and successfully completed in 96 (97%). The median age and body mass index were 46 (range 22-73) years and 28 (range 17-58) kg/m^2^, respectively. The majority (49, 51%) were aged between 30 and 50 years of age, while one-third were postmenopausal. Abnormal uterine bleeding was the presenting symptom in 40 premenopausal and 24 postmenopausal women; 26 (27%) women had infertility; seven (7.3%) had increased vaginal discharge, while 26 (96%) were asymptomatic. None had prolapsed polyps on physical examination. Often considered to be procedural challenges, nulliparity, prior cesarean births, and postmenopausal status were noted in 25 (26%), 22 (23%), and 33 (34%) women, respectively. Suboptimal imaging in two patients led to misdiagnosis of submucous fibroids as polyps, and they could not be removed with a grasper; the third unsuccessful attempt was due to failed entry in a postmenopausal woman. There were no procedure-related complications. The median procedure duration was 15 (IQR, 10-20) minutes, and the median pain score was 3 (IQR, 2-4) on a visual analog scale of 0-10 [8].

Contrary to expectations, larger polyps were easier to visualize, grasp, tug, twist, and retrieve than minuscule ones, and a transvaginal ultrasound with saline instillation was necessary to confirm completion. Saline sonography also proved useful when there was significant acoustic shadowing secondary to large uterine fibroids and adenomyosis. Softer polyps and those with multiple cystic spaces as in postmenopausal women were easily retrieved although sometimes in piecemeal.

## Discussion

**Figure 2:**
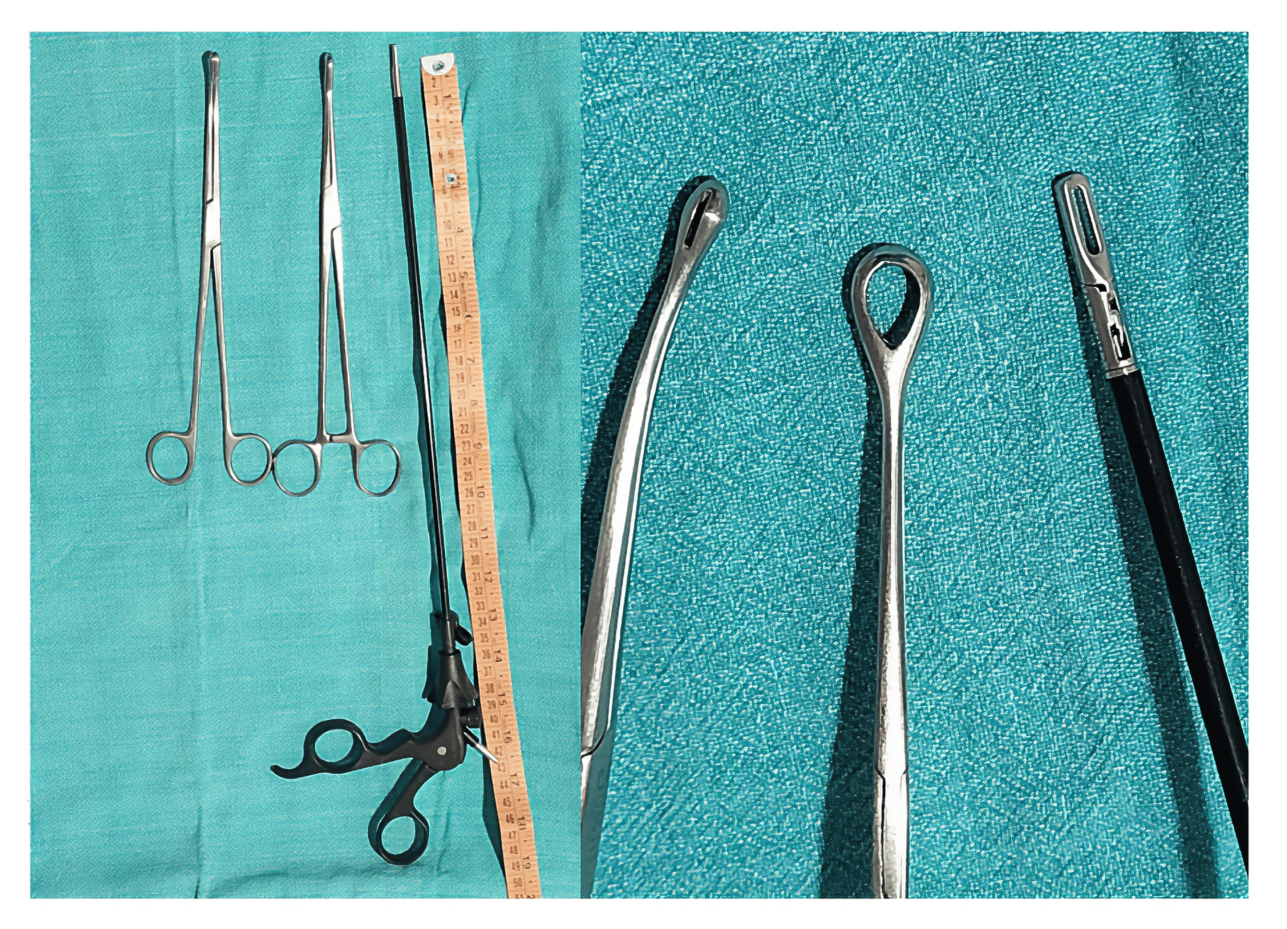
Ovum forceps, sponge-holding forceps, and laparoscopic grasper

As with any outpatient procedure, this technique offers a one-stop solution without the need for hospitalization. This is particularly beneficial in the elderly with co-morbidities in whom anesthesia with its attendant risks can be avoided, particularly if a malignant polyp is suspected, which would necessitate a subsequent definitive procedure. The potential risks with the procedure include false passage and uterine perforation, which are minimized by ultrasound guidance. The use of a laparoscopic grasper for polyp retrieval offers several advantages. Figure 2 depicts the length and reach of the laparoscopic grasper for intrauterine manipulation in comparison to an ovum forceps or a sponge-holding forceps, which are limited by the presence of the box lock. Moreover, the latter two instruments have wider jaws in comparison. The technical difficulties posed by enlarged uteri, elongated, stenosed cervices, and fundal and cornual location of the polyps are overcome to a large extent by the use of the laparoscopic grasper. Additionally, the diameter of this instrument does not necessitate cervical dilatation, thereby avoiding the resultant pain. This makes it an invaluable tool for use in nulliparous, postmenopausal women and those with prior cesarean births. Ease of availability makes it a viable option even in low-resource settings.

The available literature on outpatient endometrial polypectomies focuses on hysteroscopic techniques with very few reports of ultrasound-guided procedures. The study by Lee et al. in 2006 described transvaginal ultrasound-guided endometrial polypectomy in 37 women using a specially adapted tenaculum [5]. The procedure was successful in 86.5% of them with a mean procedure duration of eight minutes, but two patients needed cervical sutures for tenaculum-induced bleeding. In 2016, Moon et al. reported transabdominal ultrasound-guided polypectomy using a sharp curette in women undergoing in vitro fertilization [6]. They found this to be more advantageous than hysteroscopic polypectomy, as the latter can negatively affect embryo implantation due to fluid infusion, particularly with the use of large-diameter rigid scopes. Sarkar and Hochberg described ultrasound-guided removal of a 2-cm endometrial polyp using a universal grasping forceps in 2020 [9], around the same time our technique was developed. Subsequently, they published their results in 30 women, reporting an average operating time of 12 minutes and a median pain score of 5 [10]. In the present study, more than 95% of polyps could be retrieved successfully by the ultrasound-guided procedure, with comparable operating times; moreover, no procedure-related complications were noted.

One of the limitations of the present study is the lack of follow-up data. A postprocedure ultrasound examination at three months to look for residual polyps has now been incorporated into our practice. Follow-up data on residual polyps are necessary to determine the need to refine the technique. A skilled ultrasound operator is an essential prerequisite for successful completion of the procedure, as opposed to hysteroscopy, where the surgeon has the benefit of direct visualization of the uterine cavity. We did not have a comparator group as we sought to describe our initial experience with this technique. Subsequently, a study was designed to compare this with hysteroscopy under anesthesia (the standard procedure at our institute) as part of a student thesis. Prospective studies comparing its safety and efficacy with officehysteroscopy will help define the role of ultrasound-guided polypectomy in ambulatory gynecology.

## Conclusions

To conclude, ultrasound-guided endometrial polyp removal appears to be a safe procedure for the removal of endometrial polyps. It has all the inherent benefits of an office procedure. The setup is not resource-intensive, and the retrieval technique can be easily mastered, but the success is largely dependent on the expertise of the ultrasound operator. Studies from multiple centers comparing it with hysteroscopy are needed to validate the results. Data on residual/recurrent polyps after the procedure is essential for preoperative counseling. If safety and efficacy are confirmed by future studies, it can represent a cost-effective alternative to hysteroscopic polypectomy and potentially obviate the need for hysteroscopy in eligible women.
